# Occurrence and Toxicological Risk Assessment of Polycyclic Aromatic Hydrocarbons and Heavy Metals in Drinking Water Resources of Southern China

**DOI:** 10.3390/ijerph15071422

**Published:** 2018-07-06

**Authors:** Muting Yan, Huayue Nie, Wenjing Wang, Yumei Huang, Jun Wang

**Affiliations:** 1 College of Marine Sciences, South China Agricultural University, Guangzhou 510642, China; marineymt@scau.edu.cn (M.Y.); hynhy@stu.scau.edu.cn (H.N.); wenjing1379@163.com (W.W.); huangyumei@scau.edu.cn (Y.H.); 2 Joint Laboratory of Guangdong Province and Hong Kong Region on Marine Bioresource Conservation and Exploitation, South China Agricultural University, Guangzhou 510642, China

**Keywords:** drinking water, health risk, PAHs, heavy metals, water pollution

## Abstract

Polycyclic aromatic hydrocarbons (PAHs) and heavy metals exposure is related to a variety of diseases and cancer development, posing a great health risk to humans. In this study, water samples were collected from nine important water sources in Guangdong, Guangxi and Hainan provinces to determine the degree of PAHs and heavy metals contamination. Overall, the total contents of 16 PAHs and heavy metals were found within the permissible levels. In human health risk assessment, the benzo(*a*)pyrene equivalent concentration (BaP_eq_) presented a much lower level than the guideline values announced by Chinese Environmental Protection Agency (CEPA) and United States Environmental Protection Agency (US EPA), demonstrating that the PAHs contamination level in drinking water was mostly acceptable. For heavy metals, the Chronic daily intake (CDI), hazard quotient (HQ) or hazard index (HI) suggested that the water quality in nine water sources was desirable and did not present a risk to human health.

## 1. Introduction

Water pollution by Polycyclic aromatic hydrocarbons (PAHs) and heavy metals is widespread in many countries worldwide. Excessive human activities and inappropriate disposal of industrial wastes are some of the reasons that lead to their detection in important water resources, including sea water, river water and underground water, which directly affects the quality of drinking water and aquatic organisms [1].

PAHs, a large class of organic pollutants consisting of 2–6 fused aromatic rings without heteroatoms or substitutes, are the earliest known chemical carcinogens [2]. In nature, PAHs exist mainly in bitumen, tar, coal and petroleum, which are mainly enhanced by incomplete combustion of hydrocarbon containing compounds [3]. In the last two decades, PAHs in environment dramatically increased due to anthropogenic activities including exhaust gases from automobiles, aircraft, and various motor vehicles [4,5]. Surface runoff, wastewater discharge and atmospheric deposition are the major ways that PAHs flow into water bodies [6]. As a potent carcinogen, short-term and long-term exposure to PAHs could damage the immune, reproductive, hematopoietic and nervous systems, as well as affect overall human health [3]. Thus, PAHs have attracted worldwide attention and 16 of them were listed as priority pollutants by US EPA [7,8]. With the development of industry, PAHs have also been identified in drinking water resources collected from several major water systems of China, including Yellow River [9], Qiantang River [10] and Pearl River [11], where concentrations generally exceed the US EPA guideline value of 200 ng/L. Drinking water is closely related to human health its safety should be ensured. However, trace amounts of PAHs in water are difficult to remove through conventional water treatment in China. Thus, it is necessary to evaluate the PAH contamination status in drinking water resources of southern China to protect human health [3,10].

Heavy metals are common pollutants in the environment. They are stable, non-biodegradable and accumulative, making them a global problem [12]. Both anthropogenic activities and natural actions contribute to heavy metal abundance in the environment [13,14,15,16]. Solid waste heaps; disposal of metals; agricultural, municipal and industrial waste; animal and human excreta; geological weathering; and leaching of metals from garbage are the main sources of environmental heavy metals [17,18]. Although some heavy metals are necessary for biosynthesis in human, excessive amounts may be toxic, such as nickel (Ni), copper (Cu) and zinc (Zn). Some metals have adverse effects on human even at very low concentrations, including lead (Pb) and chromium (Cr) [19,20]. Due to their toxicity and non-biodegradability, the discharged heavy metals in water seriously threaten aquatic organisms and human health via drinking water as well as food chain pathways [21]. Heavy metal pollution in drinking water is becoming an increasing problem globally. High concentration of Pb was detected in drinking water of Bandar Sunway in Malaysia [22]. In Uganda, concentrations of Cu, cobalt (Co) and Ni in household water samples exceeded UK drinking water thresholds of 200 μg/L [23]. Reports have also shown that high concentrations of Arsenic (As), selenium (Se) and thallium (Tl) were detected in drinking water around China [24].

Drinking water resources are the basis of human existence, which should be safe and unpolluted. Previous studies on PAHs and heavy metals in China mostly focused on their accumulation, transfer and source in wastewater-irrigated soils, especially in North China. There are few studies on PAHs and heavy metals in drinking water resources in southern China [25,26,27]. Major rivers in China were reported to show significantly higher concentrations of pollutants than those in Europe, Australia and the USA [28]. Thus, it is of great importance to investigate the pollution status of PAHs and heavy metals in drinking water resources of southern China. In this study, heavy metals and PAHs concentrations were detected in nine important water sources from Guangdong, Guangxi and Hainan provinces. The potential pollutant sources were identified and a risk assessment was conducted to evaluate the risk relative to PAHs and heavy metals occurrence in drinking water resources in southern China.

## 2. Materials and Methods

### 2.1. Sample Collection

Water was sampled from nine resources and reservoirs: Longtang and Chitian reservoirs in Hainan province; Pinggang and Dongjiang tributary water resources in Guangdong province; and Yongjiang, Xunjiang, and Liujiang water resources, Suyan reservoir and Guilin waterworks in Guangxi province. All of them are important drinking water resources in southern China, providing about 5170,000 m^3^ drinking water per day for more than 18.89 million people. Table 1 show the sampling regions and details. Clean 2.5 L bottles were used to collect water from the surface during June 2013. Three samples (2 L each, *n* = 27) were collected from the surface water (0–15 cm) of each site. Each sample was collected in duplicate. After sampling, bottles of water were directly transferred to laboratory and store at 4 °C prior to extraction within seven days.

### 2.2. Chemical Analysis

A mixture of PAH standards, consisting of the 16 USEPA priority PAHs (fluorene (Flur), pyrene (Pyr), fluoranthene (Fla), benzo[*k*]fluoranthene (BkF), acenaphthylene (Acy), chrysene (Chr), phenanthrene [29], anthracene (Ant), benzo[*b*]fluoranthene (BbF), dibenz[*a*,*h*]anthracene (DBahA), benzo[*a*]anthracene (BaA), acenaphthene (Acp), indeno[1,2,3-*cd*]pyrene (Ind) and benzo[*a*]pyrene (BaP), naphthalene (Nap) and benzo[*g*,*h*,*i*]perylene (BghiP)), was obtained from AccuStandard Inc. (New Haven, CT, USA).

For PAHs analysis, a solid-phase extraction (SPE) system from Supelco was used to extract water samples (1 L each sample). After washing with ethyl acetate (5 mL), 5 mL methanol and deionized water were used to condition the SPE cartridges, respectively. Then, the samples flowed into the cartridges (6 mL/min) under vacuum and eluted with ethyl acetate (3 mL). Anhydrous Na_2_SO_4_ was used to remove the water from the extracts, which were reduced by N_2_ blow-down [10].

A gas chromatograph with mass selected detector (MSD) (Agilent 5975, Agilent Technologies, Santa Clara, CA, USA) was used to analyze all samples. The chromatographic column was a HP-5 (30 m × 0.25 mm i.d. × 0.25 μm film thickness) capillary column (Agilent, USA) with highly purified helium. When the temperature of the injector was 280 °C, 1 μL aliquot sample was injected under splitless mode. The temperature of the column was set initially at 50 °C (3 min), gradually increased to 200 °C (10 °C/min) and then 280 °C (5 °C/min), finally maintained constant at 280 °C for 10 min. To determine the background contamination, field blanks were sampled at the same time. The method blanks and spiked blanks showed that no detectable PAHs in the background. Surrogate standards were used to monitor the matrix effects and procedural performance by adding to the samples [30]. The method detection limits (MDLs) were identified when a peak with a signal-to-noise ratio of 3 was observed. PAH recoveries were studied to illustrate the efficiency. For the 16 USEPA priority PAHs, the average recovery values ranged 86–118%. The relative standard deviations (RSD) were all below 12%.

For heavy metal analysis, water samples were filtered and analyzed directly. Inductively coupled plasma-mass spectrometer (ICP-MS) was applied to analyze heavy metals, including Cu, As, Zn, mercury (Hg), Ni, cadmium (Cd) [31], Pb and Cr. Each sample was analyzed three times. The operating conditions of ICP-MS are as follows: nebulizer gas (argon) flow rate, 0.9 L/min; auxiliary gas (argon) flow rate, 0.3 L/min; plasma (argon) gas flow rate, 15 L/min; reaction gas flow (helium) rate, 4 mL/min; lens voltage, 7.25 V; and ICP RF power, 1100 W. Standard reference materials used for the quality assurance and quality control (QA/QC) were from Chinese Academy of Measurement Science (GBW07401). To characterize the analytical precision, the standard reference solution was measured three times and RSD was calculated. In the standard reference material, the average recoveries of metals analyzed in the study ranged from 93% to 112%.

### 2.3. Human Health Risk Assessment

#### 2.3.1. CDI for Heavy Metals Indices

Risk assessment is used to estimate the probability of an event and the adverse health effects that may occur within a given period. CDI is the index to evaluate human body intake for heavy metals via contaminant contact [3,32,33].

The formulas are as follows:

Ingestion:

$${\mathrm{CDI}}_{i}=\frac{C\times \mathrm{IR}\times \mathrm{ED}\times \mathrm{EF}}{\mathrm{BW}\times \mathrm{AT}}$$

$$\mathrm{Dermal}\;\mathrm{contact}:\;{\mathrm{CDI}}_{d}=\frac{C\times \mathrm{SA}\times \mathrm{AF}\times \mathrm{ABS}\times \mathrm{ED}\times \mathrm{EF}}{\mathrm{BW}\times \mathrm{AT}}$$

where CDI*_i_* and CDI*_d_* are daily intake through ingestion and dermal pathway (μg/kg/day), respectively; C represents the concentration of heavy metals (μg/L); IR represents the ingestion rate of water (2 L/day); EF is the abbreviation of exposure frequency and set 350 day/year; ED means exposure duration and the value is 70 years; body weight was abbreviated as BW (65 kg); AT means averaging time (25,550 days); SA means surface area (1.6 m^2^); AF means adherence factor (37.5 L/m^2^·day); and ABS is the absorption factor (0.01).

#### 2.3.2. HQ Indices for Heavy Metals

The potential non-cancer risk for heavy metals is expressed by hazard quotient (HQ). Excessive value of HQ (a value of 1) indicates that potential health risk should be concerned. The greater is the value, the greater is the level of concern. Rfd means the reference dose, i.e., the dose that individuals can remain under for long periods without any adverse effects [34,35].

HQ = CDI/Rfd

HI = ∑HQ

#### 2.3.3. Carcinogenic Risk for PAHs

To assess the carcinogenic risk of PAHs, BaP-equivalent concentration (BaP-eq) was calculated from the toxic equivalent factors (TEFs) of the PAH relative to the carcinogenic potency of BaP, which were proposed by LaGoy and Nisbet in 1992 [36].

### 2.4. Statistical Analysis

Excel 2007 (Microsoft Office, Microsoft, Redmond, WA, USA) was used to calculate the descriptive statistics. SPSS software (version 16, SPSS, Chicago, IL, USA) was applied to the univariate and multivariate statistical analyses, including the inter-metals correlation and principal component analysis (PCA).

## 3. Results and Discussion

### 3.1. Concentration and Distribution

**Figure 1:**
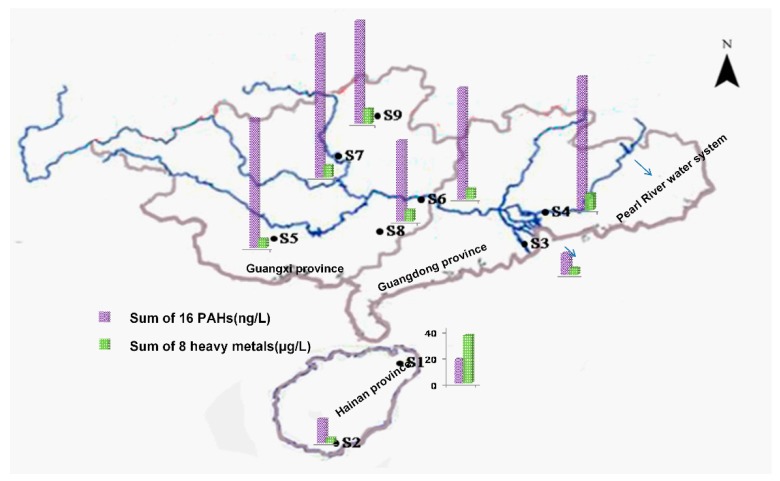
Sampling sites and concentrations of PAHs and heavy metals in the study area. The vertical axis is marked for Site 1.

Table 2 and Figure 1 show the ranges, mean and median concentrations of PAHs in samples from water resources of southern China. In all samples, only 9 of the 16 PAHs were detected. The total PAH concentrations were in the range from 16.59 to 108.91 ng/L (mean, 65.25 ng/L). The highest PAHs level was detected at Site 7 with 108.91 ng/L (the Liujiang water resource in Guangxi province), followed by Site 4 with 101.78 ng/L (Dongjiang tributary, Guangdong, China) and Site 5 with 98.80 ng/L (Yongjiang, Guangxi, China). While the lowest level was detected at Site 3 (the Pinggang water resource in Guangdong province). Liujiang reservoir is located in Liuzhou City (Guangxi, China), an important industrial base of southern China with leading industries in metallurgy, papermaking and chemical industry. The significant discharge of wastewater may increase the amounts of PAHs at Site 7. Sites 3–9, belonging to the Pearl River water system, had total concentrations that were much lower than those in their downstream, the Pearl River Delta [11]. Furthermore, the concentration of Ant was highest among the 16 priority PAHs, followed by Phe, Flur, Ind, Bap, Acp, Pyr, BaA and DBahA with concentrations of 23.88, 22.33, 21.91, 19.79, 19.73, 19.10, 16.59, and 16.51 ng/L, respectively.

To compare the PAHs levels with other water resources, PAH contamination detected in different rivers around the world are listed in Table 3. In our study, levels of PAHs were similar to the source of the three gorges reservoir (China) [37] and Japaratuba River (Brazil) [38], but much higher than those of Gomti River (India) [39] and even five times higher than the Wyre River (England) [40]. Besides, comparing the PAHs concentrations with those of Yellow River, Yangtze River and Qiantang River indicated that the PAH concentrations in the present study were somewhat low in China.

Table 4 summarizes the contamination status of heavy metals in samples collected from southern China. Based on the obtained data, the order of average concentrations in the water sources were observed as follows: Zn > As > Cr > Ni > Hg > Cu = Pb > Cd. At the nine sampling sites, mean value of total concentrations (sum of the eight heavy metals) was 8.35 µg/L, with a range from 3.44 to 36.63 µg/L. Among the tested heavy metals, Zn was the most abundant, followed by As, Ni, Cu, Hg, Pb, Cr and Cd. Concentrations of all the heavy metals were below the World Health Organization (WHO) permissible limits [21]. Among all of the samples, the highest level of Zn (31.05 µg/L), Hg (1.12 µg/L), Cr (0.436 µg/L), and Pb (0.60 µg/L) was detected at Site 1 (Longtang waterworks), and Cr was only detected at this site; the highest level of Ni (1.32 µg/L) and Cu (0.62 µg/L) was detected at Site 4 (the Dongjiang tributary); and the highest level of As (1.19 µg/L) and Cd (0.007 µg/L) was detected at Site 3 (Pinggang resource) and Site 7 (Liujiang resource), respectively.

### 3.2. Profiles and Sources

**Figure 2:**
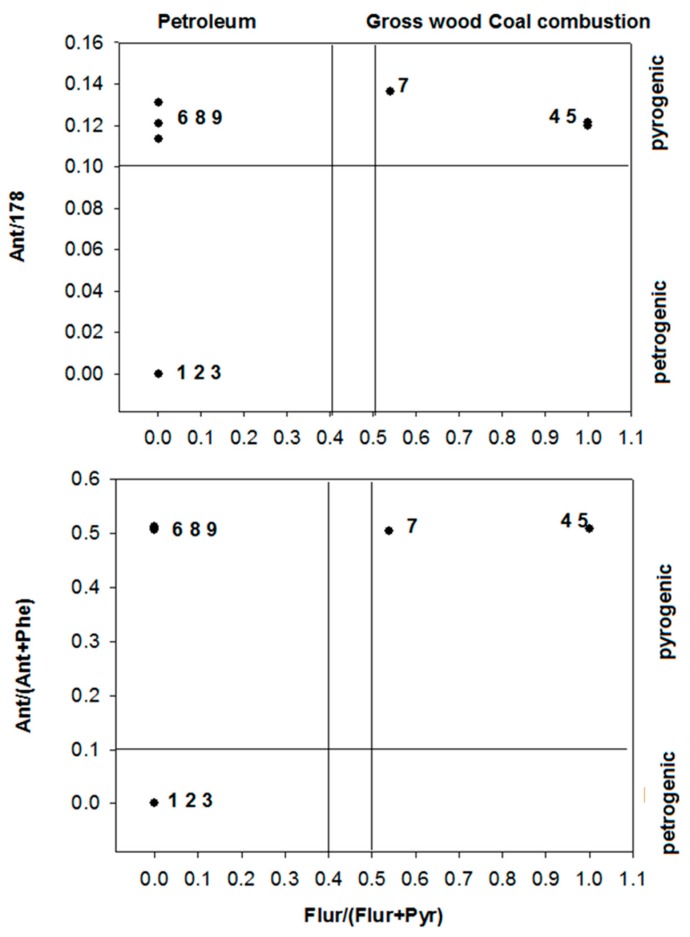
PAH cross plots for the ratios of Ant/178 vs. Flu/(Flu + Pyr) and Ant/(Ant + Phe) vs. Flu/(Flu + Pyr).

Different ratios of some PAHs can indicate the sources of pollution, including diagenetic, petrogenic and pyrogenic processes [49]. Ratios such as Ant/178, Ant/(Ant + Phe), Flu/(Flu + Pyr), FlA/(FlA + Pyr) and BaA/(BaA + Chr) are common indices for deducing possible sources [50,51]. In general, petrogenic origin results in Ant/178 and Ant/(Ant + Phe) ratios below 0.1, whereas pyrogenic processes cause higher ratios (≥0.1). Similarly, low Flu/(Flu + Pyr) ratio (<0.4) is related to petrogenic origin while ratio ≥0.5 is produced by wood, grass and coal combustion origins [52]. Ratios of Ant/178 vs. Flu/(Flu + Pyr) and Ant/(Ant + Phe) vs. Flu/(Flu + Pyr) in the water samples from southern China are shown in Figure 2. It could be concluded tha PAH pollution at Sites 1–3 originated from petrogenic possesses; pollution at Sites 4, 5 and 7 mainly generated from coal, grass and wood combustion; and PAHs at Sites 6, 8 and 9 might result from petroleum hydrocarbons.

As for the heavy metals contamination, relationship between metals provided interesting information about their sources and pathways. In all water samples, Cr, Hg, Pb and Zn, which were mainly attributed by industrial activities, revealed a strong positive correlation (*r* > 0.84). These results suggested that discharging of industry effluents might be the major sources of heavy metals contamination, such as pollutants from printing, smelting, mining, etc. Besides, Cu was strongly and significantly positively correlated with Ni (*r* = 0.88), as well as related to the concentration of Pb. As for As and Cd, and most other heavy metals, no significant correlations were observed, which might be contributed by the different behavior during transport in most areas of southern China.

**Figure 3:**
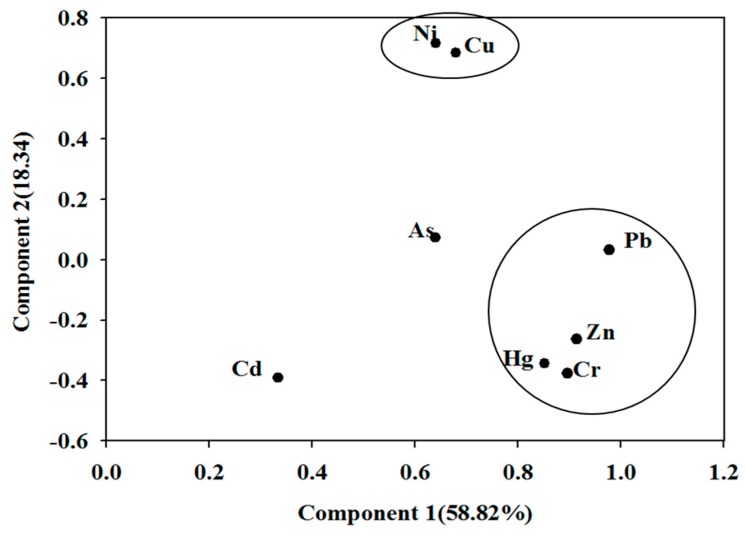
Loading plots of the first two components obtained for the concentrations of heavy metals in the water samples.

PCA, a statistical technique, is commonly applied for reducing the dimensionality of complex data sets and figuring out the key variables relative to the observed differences to preserve the relationships between the original data [53]. Orthogonal principal components (PCs) extracted via PCA are also employed to characterize the maximum variance in the analysis. In this study, two PCs accounting for about 76.16% of the total variance are presented in Figure 3. PC1 was dominated by Cr, Zn, Hg and Pb, which were significantly correlated with each other. The highest concentrations of these metals occurred at Site 1 (Longtang waterworks), as the surrounding rocks predominantly basalt, and SiO_2_, K_2_O and Na_2_O are the main components. Therefore, the source of Cr, Zn, Hg and Pb appears to be anthropogenic from traffic activities, domestic wastewater and industry. Cr might be mainly attributed by industrial activities. For a long time, high concentrations of Pb has been recognized as closely interrelated with the utilization of leaded gasoline in traffic activities [54]. In addition, wide use of composted materials, liquid manure, fertilizers and pesticides in agriculture is blamed for Zn pollution, as well as the industrial sources [55].

Another group is composed of Ni and Cu (PC2), which also had a high loading in PC1, suggesting a mixed source (natural and anthropogenic) contributed to the heavy metals pollution, including the waste released from industries and combustion of fuel. Besides, wastewater from sewage treatment plants, municipal sewage sludge and groundwater near landfill sites probably gave rise to the increased Ni in water [56]. Between the two components, Cd and As were divided almost equally, which also suggested a mixed natural and anthropogenic source. As Cd is a relatively mobile element, usage in glass, plastics, ceramics and paint manufacture mainly account for its enhanced levels in the environment [55].

### 3.3. Risk Assessment

Total BaP_eq_ calculated from the TEF values of the 16 PAHs ranged from 2 × 10^−5^ to 0.02 µg/L in our nine exposure cases. BaP_eq_ values in all of the sample sites were obviously lower than guideline values recommended by the US EPA (200 ng/L) and CEPA (2.8 ng/L) [57,58]. However, most PAHs could induce adverse effects on human health because of their toxicity and carcinogenicity, even at low concentrations. The detection of PAHs suggested contamination in these sample source areas, which should be alerted [58].

Table 5 summarizes the calculated CDI, HQ and HI values of heavy metals for ingestion and dermal absorb of drinking water. In risk assessment, drinking water and dermal absorb pathways were the major assumed routes of heavy metals exposure to human [59]. The average daily intakes of all heavy metals via ingestion were 10^3^ to 10^4^ times higher when compared with the cutaneous absorption, with a maximum of 3.21 × 10^−5^ for all heavy metals.

For different exposure pathways, HQ and HI of individual heavy metal were all below 1, which means there are minimal toxic risks caused by drinking water exposure in southern China. In addition, the HQ values of Cr, Cd, Ni, As, Cu, Zn, Pb and Hg tended to be less than those detected in drinking water collected from Kohistan region, northern Pakistan [21]. HQ of individual metal through ingestion was higher than that through dermal absorption (Table 5), denoting that ingestion is the main exposure pathway of heavy metals in drinking water resources The highest risk HI through ingestion was 3.45 × 10^−2^ at Site 1 (Longtang waterworks), followed by 2.43 × 10^−2^ (Yongjiang resource), 2.39 × 10^−2^ (Xunjiang resource), 2.36 × 10^−2^ (Pinggang resource), 2.32 × 10^−2^ (Dongjiang tributary), and the lowest was 4.86 × 10^−3^ at Suyan reservoir.

## 4. Conclusions

The contaminations of PAHs and heavy metals in the drinking water resources were relatively low in southern China. Mean concentration of 16 PAHs was 65.25 ng/L, with a range from 16.59 to 108.91 ng/L at the nine sampling sites. The highest PAH contents were detected at the Liujiang water resource in Guangxi province. All concentrations of heavy metals were found within the permissible levels set for the drinking water quality by WHO. The PAH ratios indicated Sites 1–3 had a petrogenic origin for pollution; Sites 4, 5 and 7 were contaminated by PAHs mainly due to combustion of coal, wood and grass; and petroleum combustion probably contributes to the PAHs detection at Sites 6, 8 and 9. The PCA results indicated that Cr, Zn, Hg and Pb in the studied drinking water were dominated by the traffic activities, domestic wastewater and industry. Ni and Cu might originate from natural and anthropogenic sources, such as waste released from industries or combustion of fuel. As and Cd also had a highly mixed source, but different from Ni and Cu.

In all studied regions, the BaP_eq_ concentrations presented a much lower level, compared with the guideline values set by CEPA and USEPA. For heavy metals, the CDI, HQ and HI indices for health risk assessment suggested that the water quality in nine water sources was desirable and did not present a risk to human health.

## Figures and Tables

**Table 1:** Detailed descriptions of the sampling sites.

NO.	Sample Site	Type of Water	Daily Production (m^3^)	Number of Population
S1	Longtang waterworks	Drinking water of Haikou city	290,000	1.77 million
S2	Chitian reservoir	Drinking water resource of Sanya city	150,000	0.57 million
S3	Pinggang reservoir	Drinking water resource of Zhuhai and Macau	1,000,000	2.15 million
S4	Dongjiang reservoir	Drinking water resource of Dongguan	2,400,000	6.95 million
S5	Yongjiang reservoir	Drinking water resource of Nanning	1,120,000	3.45 million
S6	Xunjiang reservoir	Drinking water resource of Wuzhou	NA	0.53 million
S7	Liujiang reservoir	Drinking water resource of Liuzhou	NA	1.40 million
S8	Suyan reservoir	Drinking water resource of Yulin	110,000	1.10 million
S9	Guilin waterworks	Drinking water of Guilin	100,000	0.97 million

NA, not available.

**Table 2:** PAHs concentrations in source water (*n* = 27) of southern China.

PAH Compounds	Range (ng/L)	Mean (ng/L)	Mid (ng/L)
Acp	nd–19.73	12.82	18.99
Flur	nd–22.33	7.06	nd
Phe	nd–23.87	14.23	20.52
Ant	nd–24.29	14.7	21.31
Pyr	nd–19.10	6.29	nd
BaA	nd–16.59	1.84	nd
BaP	nd–19.79	2.2	nd
Ind	nd–21.91	2.43	nd
DBahA	nd-16.51	3.67	nd
∑PAHs	16.59–108.91	65.25	60.82

nd—not detected; Acp—acenaphthene; Flur—fluorene; Phe—phenanthrene; Ant—anthracene; Pyr—pyrene; BaA—benzo[*a*]anthracene; BaP—benzo[*a*]pyrene; Ind—indeno[1,2,3-*cd*]pyrene; DBahA—dibenz[*a*,*h*]anthracene.

**Table 3:** List of sources of PAHs concentrations in source water and drinking water in the world.

Location	Type of Water	PAHs	PAHs Range (ng/L)	Reference	Date of Sampling
Chongqing and Hubei province, China	The three gorges reservoir	16	13.8–97.2	[37]	2008
Gansu Province, China	Yellow River	16	548–2598	[9]	2013
Guangdong Province, China	Pearl River Delta	16	92.8–324	[11]	2016
Guangxi Province, China	Underground River of Dashiwei	16	54.7–192.0	[41]	NA
Guizhou Province, China	Hongfeng Lake	16	167.1–336.4	[42]	2005
Henan Province, China	Yellow River	15	185–2182	[43]	2004
Hubei Province, China	Wuhan reach of Yangtze River	11	242–6235	[44]	2005
Jiangsu Province, China	Lake Taihu	16	45.4–232.74	[45]	2010
Shandong Province, China	Yellow River Estuary	16	8.51–402.84	[46]	2013
Yunnan Province, China	Groundwater and Kuaize River	13	58.0–275.5	[47]	2007
Zhejiang Province, China	Qiantang River	15	70.3–1844	[10]	2005–2006
England	Wyre River	28	2.7–20	[40]	2010–2011
Italy	Tiber River	16	1.75–608	[48]	2014–2015
Brazil	Japaratuba River	16	4.4–119	[38]	2016–2017
India	Gomti River	16	0.06–84.21	[39]	2004–2006

NA, not available.

**Table 4:** Heavy metal concentrations in source water (*n* = 27) of southern China.

Heavy Metals	Range (µg/L)	Mean (µg/L)	Mid (µg/L)
Cr	nd–0.44	0.48	nd
Ni	nd–0.69	0.36	0.25
Cu	nd–0.62	0.1	nd
Zn	nd–0.76	4.26	0.23
As	0.25–1.43	0.97	1.15
Cd	nd–0.007	nd	nd
Hg	0.102–1.12	0.32	0.21
Pb	nd–0.60	0.1	nd
∑HMs	3.44–36.63	8.35	11.17

nd, not detected.

**Table 5:** Summary of ingestion and dermal risk values for heavy metals in water.

	CDI*_i_*	HQ*_i_*	CDI*_d_*	HQ*_d_*
Cr	2.56 × 10^−3^	0.00–5.12 × 10^−3^	0.00–4.51 × 10^−7^	0.00–3.01 × 10^−5^
Ni	0.00–7.77 × 10^−3^	0.00–3.89 × 10^−4^	0.00–1.37 × 10^−6^	0.00–2.54 × 10^−7^
Cu	0.00–3.66 × 10^−3^	0.00–9.14 × 10^−5^	0.00–6.44 × 10^−7^	0.00–5.37 × 10^−8^
Zn	0.00–0.0182	0.00–6.08 × 10^−4^	0.00–3.21 × 10^−5^	0.00–5.35 × 10^−7^
As	1.44 × 10^−3^–8.39 × 10^−3^	4.81 × 10^−3^–2.80 × 10^−2^	2.54 × 10^−7^–1.48 × 10^−6^	2.07 × 10^−6^–1.20 × 10^−5^
Cd	0.00–4.11 × 10^−5^	0.00–8.22 × 10^−5^	0.00–7.24 × 10^−9^	0.00–1.45 × 10^−6^
Hg	5.99 × 10^−4^–6.58 × 10^−3^	4.28 × 10^−5^–4.70 × 10^−4^	1.05 × 10^−7^–1.159 × 10^−6^	3.52 × 10^−7^–3.86 × 10^−6^
Pb	0.00–3.53 × 10^−3^	0.00–9.80 × 10^−6^	0.00–6.21 × 10^−7^	0.00–6.67 × 10^−7^
HI		4.86 × 10^−3^–3.45 × 10^−2^		5.08 × 10^−6^–4.87 × 10^−5^
